# *Diaspora*, a large family of *Ty3*-*gypsy *retrotransposons in *Glycine max*, is an envelope-less member of an endogenous plant retrovirus lineage

**DOI:** 10.1186/1471-2148-5-30

**Published:** 2005-05-05

**Authors:** Sho T Yano, Bahman Panbehi, Arpita Das, Howard M Laten

**Affiliations:** 1 Department of Molecular Genetics and Cell Biology, University of Chicago, Chicago, IL 60637 USA; 2 Department of Biomolecular Chemistry, University of Wisconsin, Madison, WI 53706 USA; 3 Neuronautics, Inc., Evanston, IL 60201 USA; 4 Department of Biology, Loyola University Chicago, Chicago, IL 60626 USA

## Abstract

**Background:**

The chromosomes of higher plants are littered with retrotransposons that, in many cases, constitute as much as 80% of plant genomes. Long terminal repeat retrotransposons have been especially successful colonizers of the chromosomes of higher plants and examinations of their function, evolution, and dispersal are essential to understanding the evolution of eukaryotic genomes. In soybean, several families of retrotransposons have been identified, including at least two that, by virtue of the presence of an envelope-like gene, may constitute endogenous retroviruses. However, most elements are highly degenerate and are often sequestered in regions of the genome that sequencing projects initially shun. In addition, finding potentially functional copies from genomic DNA is rare. This study provides a mechanism to surmount these issues to generate a consensus sequence that can then be functionally and phylogenetically evaluated.

**Results:**

*Diaspora *is a multicopy member of the *Ty3*-*gypsy*-like family of LTR retrotransposons and comprises at least 0.5% of the soybean genome. Although the *Diaspora *family is highly degenerate, and with the exception of this report, is not represented in the Genbank nr database, a full-length consensus sequence was generated from short overlapping sequences using a combination of experimental and *in silico *methods. *Diaspora *is 11,737 bp in length and contains a single 1892-codon ORF that encodes a gag-pol polyprotein. Phylogenetic analysis indicates that it is closely related to *Athila *and *Calypso *retroelements from *Arabidopsis *and soybean, respectively. These in turn form the framework of an endogenous retrovirus lineage whose members possess an envelope-like gene. *Diaspora *appears to lack any trace of this coding region.

**Conclusion:**

A combination of empirical sequencing and retrieval of unannotated Genome Survey Sequence database entries was successfully used to construct a full-length representative of the *Diaspora *family in *Glycine max. Diaspora *is presently the only fully characterized member of a lineage of putative plant endogenous retroviruses that contains virtually no trace of an extra coding region. The loss of an envelope-like coding domain suggests that non-infectious retrotransposons could swiftly evolve from infectious retroviruses, possibly by anomalous splicing of genomic RNA.

## Background

Eukaryotic genomes are littered with dozens to tens of thousands of copies of reverse transcriptase (RT)-based retroelements [1-3]. Among these are a diverse collection of elements characterized by long terminal repeats (LTR) that include the *Ty1-copia*-like and *Ty3*-*gypsy*-like retrotransposon families, endogenous retroviruses, and mammalian lentiviruses [4]. LTR retrotransposons have been especially successful colonizers of the chromosomes of higher plants where they constitute as much as 80% of these genomes [3,5-7]. In soybean, several families of LTR retrotransposons have been identified [8-10], including at least two that possess an *env*-like ORF and resemble mammalian endogenous retroviruses [10,11].

The evolutionary relationship between retrotransposons and retroviruses has been well established by phylogenetic tree constructions. However, the branches linking these groups are, not unexpectedly, long ones [4,10,12-15]. The major structural difference between retrotransposon and retrovirus genomes is the presence of an envelope gene (*env*) in the latter. Retroviral envelope proteins sponsor receptor binding, cell fusion, and particle budding, and contain transmembrane and coiled-coil domains[16]. While the *de novo *acquisition of an env-like coding region by transduction could conceivably occur in a single step, the functional evolution of such a coding domain might be expected to occur over considerable stretches of evolutionary time [15,17]. But could the loss of such a coding domain occur in a single step? This question is far from implausible, considering that all retroelement genomes are RNA transcripts and many are substrates for splicing reactions. A single event of anomalous packaging of an improperly spliced subgenomic RNA, followed by reverse transcription could lead to an *env*-less element in an evolutionary blink of an eye.

In the present study, the characterization of the soybean retrotransposon, *Diaspora*, provides evidence for a relatively rapid transition between enveloped retroelements and non-enveloped retrotransposons. Our phylogenetic analysis suggests that the *Diaspora *retrotransposon emerged from a lineage of plant endogenous retroviruses that possesses an *env*-like gene [10].

*Diaspora *was initially encountered in a genomic clone as a 5'and 3'-truncated copy nested between copies of another LTR retroelement (Laten, unpublished). Using both direct sequencing and *in silico *analysis, we generated a full-length consensus copy of *Diaspora *and confirmed 1) its membership in the *Ty3-gypsy*-like family of LTR retrotransposons and 2) its status as the only member of an endogenous retrovirus lineage lacking an *env*-like gene. The *in silico *procedure can be extended to construct consensus sequences for other repetitive DNA families from degenerate elements and from single-pass-read genome survey sequences, provided the copy numbers are sufficiently high and constitute a robust collection of overlapping sequences.

## Results

### AF095730 is related to *gypsy *group LTR retrotransposons

Sequencing of subclone pAMH3C [GenBank: U96295] initially led to the characterization of an *env*-like gene and the 3' LTR of the *SIRE*1 endogenous retrovirus belonging to the *Ty1-copia *group of retroelements [11]. The DNA adjacent to the *SIRE*1 LTR constituted an initially unidentified 544 bp ORF that gave no hits in BLASTn or BLASTx searches. However, when the sequence of the adjacent subclones, pAMH3G and pAMH3D, were addended to pAMH3C and assembled into a contig [Genbank: AF095730], a single, 1383-codon ORF with a nonsense mutation at position 2604, and frameshifts at 2813 and 3139 was generated (Laten and Das, unpublished). The frameshifts occurred in runs of six thymidines and five adenosines, respectively. When the frameshifts were adjusted and the conceptual translation was used to query the Genbank protein database, numerous high scoring hits to retrotransposon reverse transcriptases and integrases were obtained (Laten and Das, unpublished).

The large collection of sequences of reverse transcriptases and integrases that were retrieved, most as contiguous polyproteins, all belonged to the *Ty3-gypsy *group of LTR retrotransposons. While the BLASTp search identified AF095730 homology to numerous accessions from residue 135 to the carboxyl terminal, no sequences with similarity to the first 134 amino acids were found. The highest scoring hits were identified as *Athila*-like, related to the *Ty3*-*gypsy *group element from *A. thaliana *[18] that has subsequently been shown to be present in a wide range of plant genomes, including soybean, other dicots, and monocots [10]. We have named the new soybean element family *Diaspora*.

### *Diaspora *is a multi-copy family

Because the *Diaspora *DNA in the original genomic clone was truncated at both ends, we initially probed the λFIXII genomic library for additional *Diaspora *copies. Hybridization detected a few thousand positive plaques, confirming the moderately high copy number of this family. DNAs from a random sample of twenty positive clones were amplified using primers derived from the ends of AF095730 (PDIA01F/PDIA02R and PDIA03F/PDIA04R). No clone produced amplicons with both primer pairs, suggesting that all copies of *Diaspora *in these clones were either 5'- and/or 3'- truncated, or polymorphic at the primer sites (data not shown). We inferred that the library would not readily yield full-length elements. Retrospectively, this finding would have been anticipated had the unusual length of *Diaspora*, approaching that of the average insert size in λFIXII, been known (see below). With the availability of BAC soybean genomic libraries with inserts in excess of 100 kb [19,20], the possibility of isolating full-length *Diaspora *copies became a virtual certainty and the λ clones were abandoned in favor of BACs. Filters containing microarrays of BAC clones derived from *G. max *cv. Forest [20] were probed for the presence of *Diaspora*. Hundreds of clones hybridized to a *prot-rt *probe (pAMH3D) and based on the number of hybridizing clones in the library, we estimated that *Diaspora *represents at least 0.5% of the *G. max *genome.

### *Diaspora *family members are truncated and heterogeneous

DNA was recovered from twenty, randomly chosen BAC clones that hybridized to pAMH3D, and PCR-amplified using the primer pairs derived from the ends of AF095730 (PDIA01F/PDIA02R and PDIA03F/PDIA04R). Surprisingly, only five clones were amplified by both primer pairs, suggesting that many copies were either 5' or 3' truncated or markedly polymorphic. Truncation would be consistent with the characterization of disrupted and nested retrotransposons first reported in maize [21]. Of those containing both termini, none successfully served as templates for more than two additional PCR-amplifications using the complete set of AF095730-based primer pairs (PDIA5F through 13R). These findings suggested that *Diaspora *is a relatively heterogeneous family. This was confirmed by limited sequencing of λFIXII [GenBank: AF095730 and AY656632-AY656653] and BAC [GenBank: AY656654-AY656662] clones using the primers listed in Table 1. A total of 15,433 nucleotides were sequenced, of which 7293 were non-overlapping. It appeared that sequencing individual members of the *Diaspora *family directly from genomic clones would not lead to satisfactory descriptions of functional coding or regulatory regions, so an *in silico *strategy for these characteristics was pursued.

**Table 1:** Primers used for PCR amplification and DNA sequencing.

Oligomer	Sequence	Oligomer	Sequence
PDIA01F	AACCTCAACAGCAAAATCAACCA	PDIA12R	CACTTTGCGAGCTGTCCTTTGA
PDIA02R	GAGGGCTGGACCATCTGAGGT	PDIA13F	TGCGGATTCACCCATTC
PDIA03F	TGGGCACATCGGACTGCTTAC	PDIA14R	CCAAAGACAACCCGATAAGGAG
PDIA04R	GACATGCCTTTCCAAAGACAACC	PDIA15F	TTCCTATCTCCTTCTTTGCTTT
PDIA05F	GGCCCAAGCAGACCATACA	PDIA16F	TTGCCCCATTGATTGCTTG
PDIA06R	TAAAAATCAACAGGGAAAATCAGT	PDIA17R	TTTCAAATCACAAAATGTCAAG
PDIA07F	TGTCTCCGCATTGATTGGTAAA	PDIA18R	TGTAAGTCAGATGGATTGCCA
PDIA08R	ATTGGCTGTCGGAGATAGGATAAA	PDIA19R	GCTCCAAGGTCCATCACGA
PDIA09F	AAACCAGTAAGACAGCCACAGAGA	PDIA20R	GGACATCCTCATCAGGGTATTG
PDIA10R	CAAGGACAGCCCCCAATG	PDIA21F	CATGGGTGCTTTGAGGGTAA
PDIA11F	GAGGTGCGATCTTTTCTTGGTC		

### *Diaspora *sequences recovered by BLASTn queries

Prior to the initiation of plant genome sequencing projects, AF095730 was used to search Genbank for related sequences. At that time, BLASTp searches returned a sizeable collection of previously characterized pol polyproteins from *Ty3*-*gypsy*-like retrotransposons (Laten, unpublished). As more and more soybean BAC-end sequences were deposited in the GSS database [22]; J. Shultz, K. Meksem, J. Shetty, C. Town, H. Koo, J. Potter, K. Wakefield, H. Zhang, C. Wu and D. Lightfoot, unpublished] the growing robustness of our BLASTn results made it clear that *Diaspora *was a high copy-number retrotransposon and that the database hits derived exclusively from BAC ends might be assembled into a contiguous, full-length, consensus *Diaspora *sequence.

Genbank sequences retrieved using sequentially selected segments of AF095730 as queries were assembled into an expanding contig. The BAC-end sequences ranged from 400 to 900 nucleotides in length. Hits with bit scores ≥200 were added to the contig and this cutoff value generated a manageable collection of sequences. Sequences anchored to the ends of AF095730 were used to extend the consensus beyond the 5' and 3' ends of AF095730 and the assembly of an expanding contig was launched. Primers generated from these flanking regions (PDIA15F through 21F) were also used to amplify and sequence additional regions from two of the full-length Diaspora candidates in BAC clones.

Two hundred seven Genbank accessions, including thirty submissions from the present study, totaling 141,423 nucleotides were collected to generate the contig. To avoid bias, duplicate sequences from different accessions were purged from the alignment. There were only three positions for which a strict consensus nucleotide could not be assigned.

**Figure 1:**
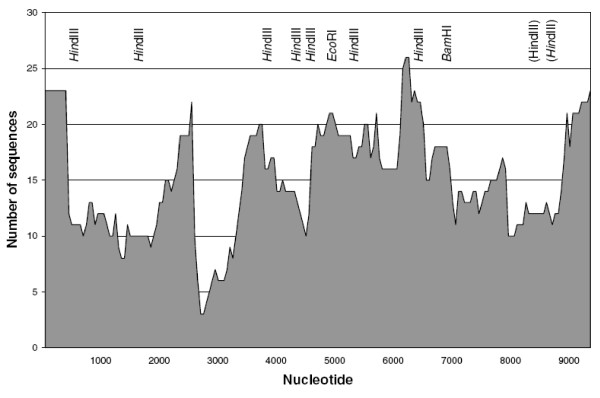
Histogram of local densities of Genbank Accessions used to construct a *Diaspora *consensus contig. Recognition sites of restriction enzymes used to generate BAC libraries are indicated. Restriction sites in () are found in < 50% of sequences. The right LTR is not shown. The contig was assembled from the following Genbank Accessions: AF095730 (this study), AY656632-AY656656 (this study), AY656659-AY656662 (this study), AQ989187, AQ989208, AQ989232, AQ989271, AQ989295, AZ044709, AZ045083, AZ221405, AZ301361, AZ302029, AZ536637, AZ933330, AZ936131, BE611677, BH000863, BH000924, BH001187, BH023628, BH023632, BH023632, BH173556, BH405523, BH405626, BH405659, BH405669, BH610143, BH610157, BH610193, BH840834, BH854486, BH888573, BH897988, BH912698, BI974271, BU546431, CC062189, CC062259, CC062269, CC062279, CC062321, CC062333, CC062399, CC062412, CC062425, CC062501, CC062524, CC062576, CC062745, CC062865, CG811196, CG812831, CG813036, CG813244, CG813336, CG813336, CG813447, CG813495, CG813591, CG813669, CG813710, CG813854, CG813944, CG814001, CG814027, CG814297, CG814428, CG814537, CG814691, CG814705, CG814739, CG814773, CG814814, CG814837, CG814944, CG814960, CG815296, CG815349, CG815376, CG815566, CG815593, CG815931, CG815990, CG816077, CG816195, CG816437, CG816499, CG816820, CG816902, CG816924, CG816965, CG817175, CG817237, CG817248, CG817294, CG817426, CG817444, CG817647, CG817665, CG817749, CG817754, CG817777, CG817807, CG817873, CG817996, CG818405, CG818428, CG818443, CG818626, CG818673, CG818711, CG819087, CG819204, CG819222, CG819552, CG819604, CG819672, CG819766, CG819790, CG819813, CG819936, CG819977, CG820067, CG820103, CG820158, CG820299, CG820411, CG820560, CG820627, CG820654, CG820656, CG820670, CG820673, CG820702, CG820718, CG820816, CG820848, CG820850, CG820868, CG821026, CG821085, CG821093, CG821150, CG821179, CG821206, CG821219, CG821294, CG821311, CG821532, CG821597, CG821693, CG821710, CG821772, CG821963, CG822140, CG822195, CG822264, CG822361, CG822369, CG822426, CG822466, CG822466, CG822582, CG823113, CG823202, CG823294, CG823320, CG823499, CG823505, CG823511, CG823713, CG824266, CG824332, CG824372, CG824380, CG824407, CG824533, CG825062, CG825163, CG825591, CG825777, CG825811, CG825933, CG826013, CL867862, CL8811208, CL881708, CL882298, CL886562, CL891285, CL899081

Fig. 1 is a histogram of the density distribution of sequences used to generate the contig. The average coverage across the length of the contig was 14.8 accessions, although inclusion of many additional sequences that met the scoring criterion in regions of high sequence conservation was not pursued. Because the initial soybean BAC libraries were created by *Eco*RI, *Hin*dIII, or *Bam*HI digestion, the local robustness of the assembly was dependent on the density of these sites in *Diaspora*. Sub-families that lacked a particular cleavage site would be under-represented. In contrast, the assembly of regions far from these sites was made possible by sub-families with additional recognition sites for one of these enzymes.

### Structure of *Diaspora*

**Figure 2:**
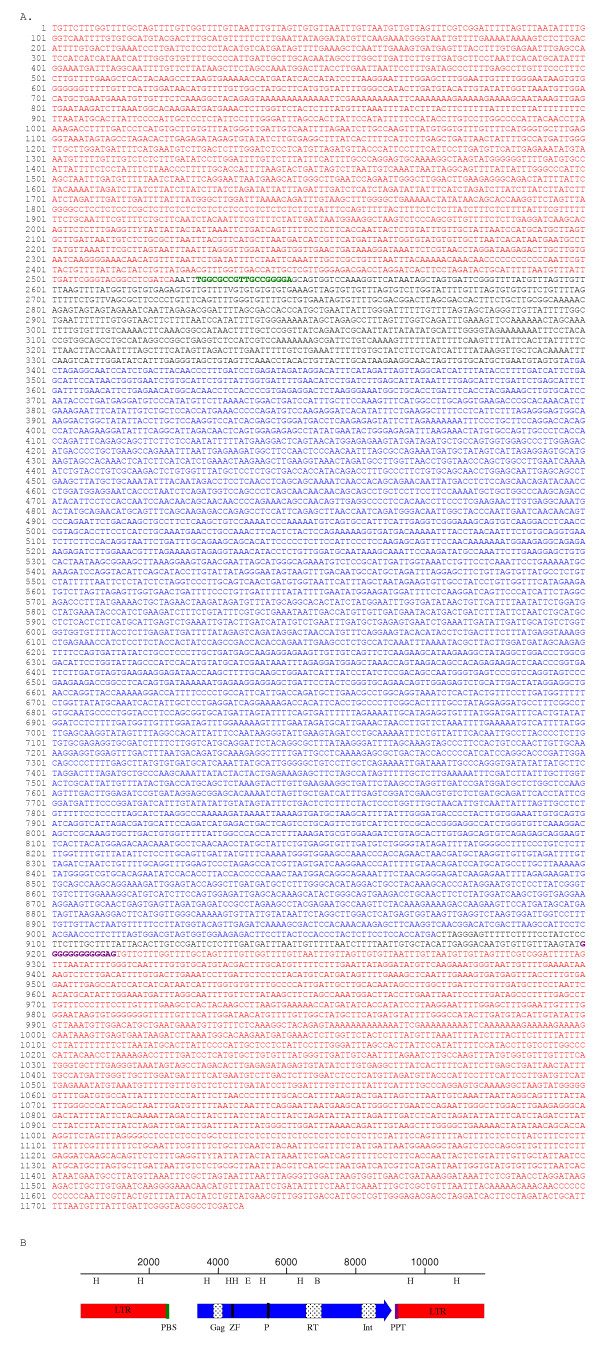
A. Consensus nucleotide sequence of *Diaspora*. LTR in red, PBS in green, ORF in blue, PPT in maroon. B. Structural organization of *Diaspora*. PBS: tRNA primer binding site; Gag: Gag core domain (CDD17379); Z: CCHC Zn finger domain; P: protease catalytic core; RT: reverse transcriptase core domain (CDD16610); Int: integrase core domain (CDD25582); PPT: polypurine tract. (⇨) ORF. Consensus restriction sites as in Fig. 2 H: *Hin*dIII; E: *Eco*RI; B: *Bam*HI.

**Figure 3:**
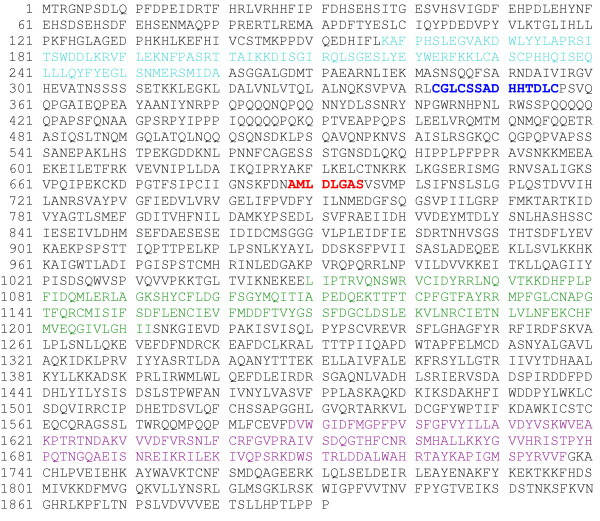
Conceptual translation of the *Diaspora *ORF. Teal: Gag core domain; blue: Zn finger domain; red: protease catalytic core; green: RT core domain; violet: integrase core domain

The length of the *Diaspora *consensus is 11,737 bp (Fig. 2), far longer than all but a handful of retrotransposons. The exceptional length of *Diaspora *is due primarily to the unusual length of its LTRs and the long gap between the upstream LTR and the *gag *start codon (Fig. 2). Like nearly all other retroelements, the LTRs terminate in TG...CA. The element is characterized by a contiguous 1892-codon ORF whose conceptual translation yields a single gag-pol polyprotein (Fig. 3) characteristic of *Ty3-gypsy*-like retrotransposons [23]. Not surprisingly, the consensus contains neither nonsense codons nor frameshifts. This translated ORF possesses core domains for gag (CDD17379), reverse transcriptase (CDD16610) and integrase (CDD25582). There is also a CX_2_CX_4_HX_4_C zinc finger motif in gag and a conserved protease catalytic domain motif, AMLDLGAS (Fig. 3). Interestingly, the first thirty amino acids of the translated ORF are not similar to the amino termini of any gag proteins in Genbank. Similarities to several gag proteins begin at position 31. Translation of the other five reading frames yielded no lengthy ORFs nor any similarities to any sequences in BLASTP searches.

**Figure 4:**
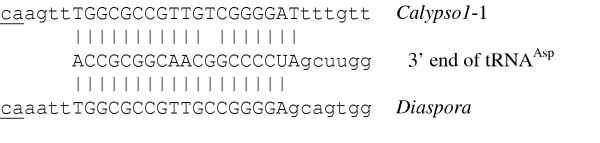
Base pairing of PBS (upper case) from *Diaspora *and *Calypso *with the 3' end of tRNA^Asp^. LTR terminus underlined.

As in the *Calypso *group [10], the tRNA primer binding site (PBS) begins 5 bp beyond the 3' end of the LTR and is perfectly complementary to the 3' terminal 18 bases of tRNA^Asp ^from *Glycine max *[24] (Fig. 4). At 873 bp, the distance between the LTR and the putative *gag *start codon is unusually long and not shared by related elements. This region contains no extended ORFs and neither BLASTn nor tBLASTn searches of the nr database retrieved significant hits.

Five potential splice donor sites, all in the LTR between 1400 and 2200 bp upstream of the *gag-pol *ORF, were predicted with medium confidence and two potential acceptor sites flanked the start codon. Although without splicing, the 5'UTR of any *Diaspora *transcript would be exceptionally long, the biological relevance of these sites is not known, and there are no reported examples of introns upstream of *gag *for any retrovirus or LTR retrotransposon.

The *pol *stop codon is 128 bp upstream of the polypurine tract (PPT) that abuts the 3' LTR. Thus *Diaspora *contains no envelope-like coding sequence beyond *pol *unlike those reported for its closest relatives, including members of the *Athila *and *Calypso *families (Wright and Voytas, 2002), *BAGY-2 *(Vicient et al., 2001), and *Cyclops-1 *[8,10,13]. When this short region was used in BLASTn and tBLASTx searches, no additional sequences with significant probabilities were recovered. Interestingly, translation of this short region yields a strongly predicted transmembrane domain, although it is interrupted by two stop codons (data not shown).

The *Diaspora *LTR is 2524 bp in length (Fig. 2), making it one of the longest among retrotransposons and contributing to its unusual length. By comparison, the *RIRE3 *LTR is 2316 [25], *BARE-*1 is 1829 [26], *Athila*1-1 is 1539, and *Cyclops*-1 is 1504 [8]. Only the LTRs from *Ogre *and *BAGY-1*, at over 5,000 and 4200 bp, respectively [27,28], are longer.

The length of the *Diaspora *LTR made it impossible to construct unique 5' or 3' LTRs by the *in silico *method employed. In addition, the absence of a contiguous element prohibited characterization of target site duplications. However, we identified eight accessions from the database that contained the tRNA PBS and part of the adjacent upstream LTR. The longest of these extended 491 bp upstream of the PBS. Twenty-two sequences contained the PPT and part of the adjacent downstream LTR. The longest of these extended 596 bp into the LTR. Thus, the central 1437 bp could not be uniquely assigned to either LTR. As a consequence, the available LTR sequences were merged to generate a single, consensus LTR that was affixed to both *Diaspora *ends.

Thirteen LTR sequences were 5' junctions and sixteen were 3', based on the complete absence of sequence similarity beyond the 5' or 3' ends, respectively, of the aligned LTR sequences. When the flanking DNAs of these 29 sequences, were used in BLASTn searches to query the GSS database, all but three generated dozens of hits (data not shown), and thus constituted repetitive elements themselves. Of the repetitive flanking DNAs, 75% represented *Diaspora *insertions into the coding regions of other retrotransposons. In addition, insertions into the coding regions of transposons related to En/Spm and Tam3 were also found. The identity of the three low- or single-copy sequences could not be ascertained. The *Diaspora *family therefore appears to be embedded in retrotransposon and transposon-rich regions. We have made similar observations for the *SIRE1 *retroelement (unpublished). Searches focused on the region upstream of the PPT failed to uncover any *Diaspora *copies with additional DNA between *pol *and the PPT.

Among the sequences used to assemble the contig, non-coding regions contained a variety of short indels, especially in homonucleotide runs and dinucleotide repeats, presumably from replication slippage. The sequences in the GSS collection from which the consensus was built represented unedited submissions, and excluding single base indels that might have been the result of unedited miscalls, most of the indels in the ORF retained the correct reading frame. Eight accessions: BH023632, CG813336, CG820702, CG821179, CG822466, AY656639, AY656648, and AY656656, were chimeric and probably represented truncated copies. All but two of these sequences were within *gag *or the putative 5'UTR. Since chimeric sequences in GSS would invariably produce lower bit scores, they were generally excluded form the contig. Consequently, many of the slightly lower scoring sequences initially retrieved in our BLASTn search were also chimeric, but were not retained for the assembly and were not further characterized.

### *Diaspora *is phylogenetically related to plant endogenous retroviruses

**Figure 5:**
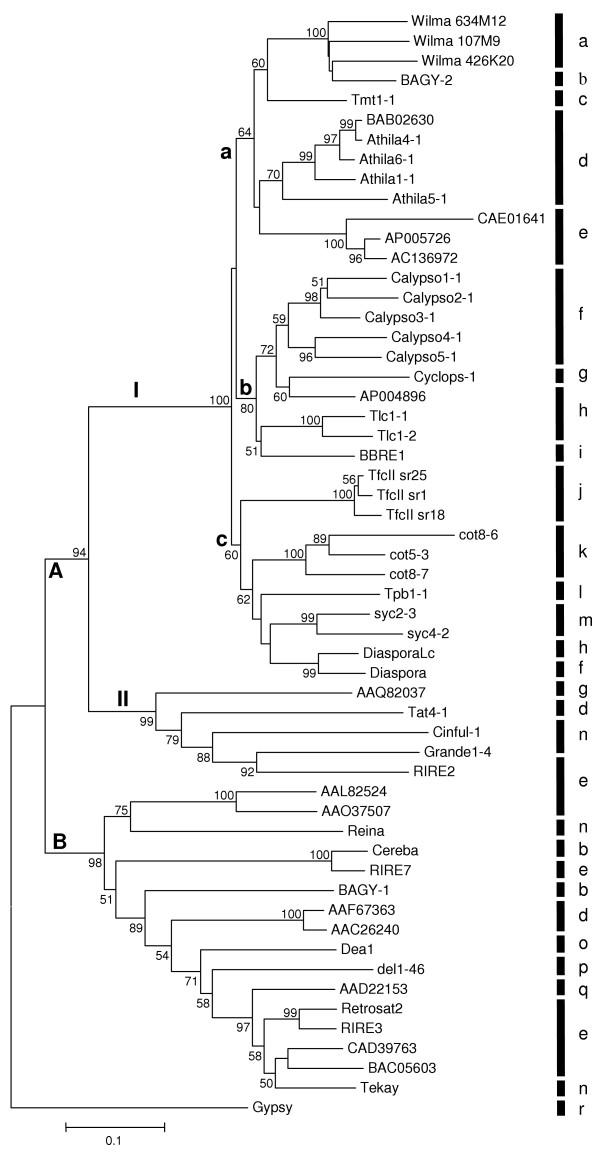
Neighbor-joining phylogenetic tree using p-distances based on conserved RT domains 2 through 7 [29] of *gypsy*-like LTR retroelements from higher plants. The tree is rooted to *gypsy*. Bootstrap values from 1000 pseudo-replicates shown as percentages only at nodes with > 50% support. Vertical line indicates genus; key below. Named elements followed by Genbank Accession numbers; unnamed elements designated by Accession Number and, for translated nucleotide sequences, first nucleotide position. Wilma 634M12: AY494981 [51]; Wilma 426K20: AY146588 [51]; Wilma 107M9: AY368673 [51]; BAGY-2: AJ279072 [13]; Tmt1-1: AC146683 (115510-125622); Athila4-1: AC007209 [10]; Athila6-1: AF104920 [10]; Athila1-1: AB005248 [52]; Athila5-1: AF147260 [10]; AP005726: 133249; AC136972: 155124; *Calypso*2-1: AF186183 [10]; *Calypso*3-1: AF186185 [10]; *Calypso*5-1: AF186186 [10]; *Calypso*4-1: AF186185 [10]; Cyclops-1: AJ000639 [8]; AP004896: 78828; Tlc1-1: AP006432 (23839-35862); Tlc1-2: AP006350 (29612-19200); BBRE1: T12085; TfcII sr1: AF219199; TfcII sr25: AF219208; TfcII sr18: AF219207; cot8-6: AF378037 [10]; cot5-3: AF378037 [10]; cot8-7: AAL06412 [10]; Tpb1-1: AC149297 (90224-102138); syc2-3: AF378052 [10]; syc4-2: AF378053 [10]; *Diaspora*Lc: AP007806: 43868; Tat4-1: AB005247 [44]; Cinful-1: AF049110 [45]; Grande1-4: X97604 [46]; RIRE2: AB030283 [47]; Reina: U69258 [48]; Cereba: AY040832 [49]; RIRE7: BAA89466 [50]; RIRE7-2: AL731604 (96205-102279); Dea1: T07863 [51]; del1-46: X13886 [52]; BAGY-1: Y14573 [27]; Tekay: AAL59229 [53]; RIRE3-2: AC123974 (48149-59938); RIRE3: AB014738 [50]; Retrosat2: AAM74400; Retrosat2-2: AL662955 (58578-70224); Gypsy: P10401 [54]. ^a^*Triticum*; ^b^*Hordeum*; ^c^*Medicago*; ^d^*Arabidopsis*; ^e^*Oryza*; ^f^*Glycine*; ^g^*Pisum*; ^h^*Lotus*; ^i^*Vicia*; ^j^*Fritillaria*; ^k^*Gossypium*; ^l^*Populus*; ^m^*Platanus*; ^n^*Zea*; ^o^*Ananas*; ^p^*Lilium*; ^q^*Sorghum*; ^r^*Drosophila*

The conserved region of *RT *was translated and the region representing peptide domains 2 though 7 [29] was used in a BLASTp search to retrieve closely related accessions from Genbank. All of the sequences retrieved were from higher plants and their distribution among species reflected, to a large extent, the current progress of genome sequencing projects. The sequences were aligned (see Additional file 1) and a neighbor joining tree was generated (Fig. 5) and was rooted to the RT from *gypsy*.

**Figure 6:**
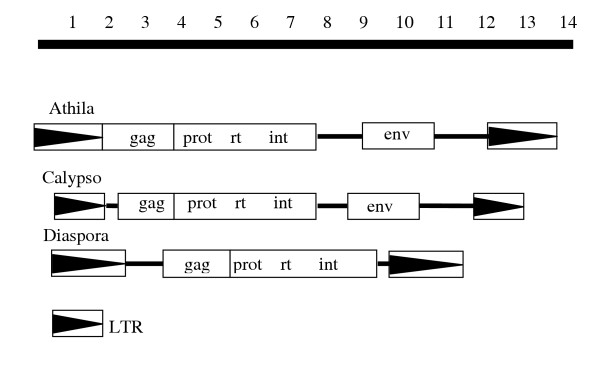
Structural organization of *Athila*, *Calypso*, and *Diaspora *consensus elements.

The tree resolves two major clades, designated A and B (Fig. 5). With respect to coding potential beyond *pol*, clade B members have none, and in all cases, the *pol *stop codon is closely followed by a PPT and the LTR. In contrast, with the exception of *Diaspora*, all members of clade A for which sequences downstream of *pol *are available contain a putative *env*-like pseudogene. The major structural difference between *Diaspora *and other members of this group is illustrated in Fig. 6. Clade A is further partitioned into sister clades AI and AII with 94% bootstrap support. Clade AI is further divided, with 100% bootstrap support, into AIa/b and AIc. The bifurcation of AIa and AIb in Fig. 5 is only weakly supported (40%) and may not be significant. Clades AIa and AIb are populated exclusively with *env*-containing members, including the Athila and *Calypso *families. The only full length members of AIc are *Diaspora*, *DiasporaLc*, and Tpb1-1. The other members of the AIc lineage are sequences from PCR-amplified *rt *fragments from sycamore and cotton [10], and three representative *rt*-containing genomic clones from *Fritillaria*. DNA downstream of the *Fritillaria rt *has not been characterized (C. Baysdorfer, personal communication). In contrast to *Diaspora *in *G. max *and its close relative in *L. corniculatus*, 1479 bp separate the *pol *stop codon from the LTR in Tpb1-1. While this region contains no identifiable or extended ORFs, there is a proximal 19-codon ORF whose conceptual translation is predicted with very high confidence to be a transmembrane domain (data not shown). There is also a 47-base polyA segment in the middle of this region, suggesting the interval contains an integrated cDNA. Thus, the AIIc lineage is not monophyletic for the absence of a long *pol*-LTR interval, but whether this region in Tpb1-1 represents a degenerate *env *cannot be determined.

In conclusion, the nesting of clade AIc within a much larger group of elements with an *env*-like gene suggests that at least *Diaspora *suffered a complete and nearly precise loss of a coding region, rather than failed to acquire one. To more exhaustively search for other members of this group, the RT from members of lineage AIc were used in tBLASTn searches. All hits, however, were already in the tree.

## Discussion

The DNA sequence of the previously unreported *Diaspora *retrotransposon was created by a combination of experimental and *in silico *methods utilizing *Glycine max *sequences currently available as single-pass-read accessions in public databases. To date, the publicly available *G. max *sequence collections, including the NR and HTGS databases, contain no full-length copies of this element. Consensus sequences for transposons and retroelements have frequently been generated from alignments of multiple family members [30-35], but the construction of a full-length consensus sequence of a new element from large numbers of short overlapping fragments has not. While appropriate for early stage genome projects like that of soybean, resorting to such a strategy is neither required nor efficacious in genomes that have been extensively sequenced, like those of *Arabidopsis*, rice, *Drosophila *and humans.

*Diaspora *has a single uninterrupted ORF encoding gag, protease, RT, and integrase as a single polyprotein. Consensus assemblies for *Athila *and *Calypso *elements also contain a single ORF for these proteins [10]. While we cannot infer that functional copies of *Diaspora *still exist in the *G. max *genome, the density of the contig assembly and the presence of a strongly conserved consensus nucleotide at virtually every position of the assembly supports the argument that a reasonable facsimile of a past functional element is depicted.

The *Diaspora *family is also present in the *Lotus corniculatus *genome, where we discovered an apparently 5'truncated copy on a Phase I HTGS clone, AP007806. Excluding indels, the lotus sequence shares approximately 80% nucleotide identity with the *Diaspora *consensus sequence over a length of 7 kb. Most of the indels in the coding region are in-frame. Like *Diaspora*, the lotus element lacks an *env*-like region. With the exception of two 7-bp and one 15-bp indels, the short intervals between the *pol *termination codon and the LTR are 88% identical between the two. Additional truncated copies of *Diaspora *family members are present on ten other Phase I HTGS clones from *L. corniculatus*.

*Diaspora *is unusual in several respects. 1) It has unusually long LTRs. 2) At 873 bp, the distance between the LTR, which should contain the promoter and transcriptional start sites, and the *gag *start codon is far longer than every other characterized retroelement except one. And 3) *Diaspora *is the only characterized envelope-less member of a lineage of plant *gypsy*-like endogenous retroviruses.

The significance of the extended length of the LTRs found in *BAGY-1 *[28], *Ogre *[27], *RIRE3 *[25], and *Diaspora *are difficult to ascertain since virtually nothing is known about the biology of these elements. The same is true for the unusually long regions between the LTR and the *gag *start codon in *Diaspora *and *Ogre*, although in the case of *Ogre*, this region contains a 550-codon ORF whose conceptual translation yields a polypeptide of unknown function. Since transcriptional start sites are always found at the U3-R junction of the LTR, an exceptionally long 5'UTR would result unless splicing occurred. However, there have been no introns reported upstream of *gag *in any LTR retroelement, including *Ogre *[27], for which transcripts have been characterized,.

Unlike all other characterized members of an apparent plant endogenous retrovirus lineage [10], *Diaspora *lacks an envelope-like coding domain downstream of *pol*. Few members of this lineage contain functional *gag-pol *genes, based on the presence of nonsense and frameshift mutations, and none contain a functional *env*-like gene based on these same criteria. The only other fully sequenced member of clade AIIc, Tpb1-1, contains a strongly predicted TM just downstream of the pol stop codon, but is contaminated by an apparent retrogene, and the region could not be characterized as *env*-like based on amino acid similarity.

The hypothetical env-like proteins exhibit little primary sequence similarity, and only those found in *Calypso *and Cyclops-2, which share 29% amino acid identity, appear to be homologous [10,12]. Without significant sequence similarity, we are reluctant to speculate whether the predicted transmembrane domain for the translated 128 bp fragment between *pol *and the PPT in *Diaspora *reflects a vestige of an *env*-like gene.

The tree in Fig. 5 is similar to that generated by Wright and Voytas [10]. In their study, many members of the endogenous retrovirus lineage were derived from *rt*-delimited PCR amplicons, few of which are included in on our tree because the presence of an *env*-like region was not empirically determined. Our analysis, however, includes several additional full-length elements whose *env*-like status has been determined. We infer from this analysis that an ancestral *Diaspora *element suffered a deletion of this region. Whether Tpb1-1 suffered a similar fate but subsequently acquired a retrogene is open to speculation. Using this region to query Genbank in BLASTn and tBLASTx searches yielded no hits. While envelope capture by LTR retrotransposons has been credited with the creation of infectious retroviruses [15,17], only the *env *genes of invertebrate elements have been phylogenetically linked to unrelated viruses [14]. The failure to uncover an analogous linkage in retroviruses has been attributed, in part, to accelerated divergence promoted by host-induced immune responses that fuel positive selection for envelope variants [14].

Studies focused on envelope loss have not been reported, although phylogenetic relatedness between mammalian retroviruses and endogenous retroviruses with *env *pseudogenes is recognized [36]. Although the *env *genes of most human endogenous retrovirus (HERV) families are marked by frameshifts, nonsense mutations, and deletions [37], in only one family, HERV-L, are all vestiges of the *env *gene lost [37]. In the case of HERV-L, the region between *pol *and the LTR is occupied by a dUTPase coding domain [37]. Other members of the class III HERV clade that contains HERV-L, including HERV-S, contain *env *pseudogenes [36]. Interestingly, with a copy number of 575, the HERV-L family is second only to the HERV-H family in abundance [36].

*Diaspora *and *Diaspora*Lc possess no trace of the *env*-like genes that are present in all members of clades AIa, AIb, and AII (Fig. 5). Whether other members of clade AIc, from cotton (*Gossypium*), sycamore (*Platanus*) and lily *(Fritillaria*), also lack an *env *region is not known, and the precise node within this clade that represents envelope loss cannot be assessed.

One explanation for an abrupt and complete loss of *env *is anomalous splicing of a genomic transcript containing *gag-pol-env*. Retroelement genomes are packaged as genomic RNA transcripts and retroviral transcripts destined for translation are often substrates for a complex pattern of splicing [38]. Alternatively, illegitimate recombination could lead to DNA loss and has been proposed as a major component of element elimination from plant genomes [39-41]. Many of the individual sequences that made up the contig contained short deletions not shared by others. This was especially true in non-coding regions (data not shown). Whatever the explanation, *Diaspora *appears to be an example of a retrotransposon that evolved from an endogenous retrovirus.

The nature of selective forces, if any, that might drive the loss of an *env *gene is open to speculation. *Env *genes, required for retroviral infectivity, are not thought to be required for retrotransposition, and it is possible that for some retroelements the gene or its protein product might attenuate the process, promoting selection for their inactivation, but with concomitant loss of infectivity. As noted above, in one of the largest families of HERV, the env gene has been replaced with a dUTPase. In plant genomes, however, the copy numbers of both putative endogenous retroviruses like *SIRE1 *[11], *Calypso*[10], and BAGY-2 [13] and retrotransposons like BARE-1 [42], Opie-2 [21], and *Diaspora *reach into the thousands. On the other hand, *env *genes in both mammalian and plant endogenous retroviruses are far more degenerate than those in *pol*, suggesting they are far less sensitive to purifying selection. The proliferation of one retroelement form or the other may be the result of random mutation and genetic drift. Nonetheless, retrotransposition, with or without an *env *gene, has been a far more successful long term reproductive strategy than retroviral infection.

## Methods

### DNA isolation, amplification, and sequencing

DNA containing the *SIRE1 *endogenous retrovirus was recovered from a λFIXII soybean genomic library (Stratagene) by standard plaque hybridization [43], and *Hin*dIII-digested fragments were sub-cloned into pSPORT1 (Life Technologies) as described [11]. DNA from three contiguous subclones, pAMH3C, pAMH3G, and pAMH3D were isolated and sequenced as described [11]. The junctions and contiguity of these subclones were confirmed by direct sequencing of the intact λFIXII genomic clone across the *Hin*dIII junctions. These sequences were previously deposited [Genbank: U96295 and AF095730]. A BLASTp search with the conceptual translation of AF095730 (see below) indicated that this accession contained the *pol *region of an uncharacterized retrotransposon. Several additional positive clones from this library were recovered and segments of the isolated DNAs were sequenced directly or amplified using Taq DNA Polymerase (Promega). For amplifications, reactions were preheated for 3 min. at 94°C, then 30 cycles were run at 94°C for 30 sec., 54°C for 30 sec., and 72°C for 1 to 2 min. Amplicons were spin column-purified (Qiagen) and sequenced as described [11]. Sequences were deposited [GenBank: AY656632-AY656653].

pAMH3D, compromising the protease and RT coding domains, was used to probe a soybean BAC library [20] (generously provided by K. Meksem) under moderate stringency [43] for the presence of sequences related to AF095730. Ten clones were chosen arbitrarily for amplification and sequencing. DNAs from BAC clones were recovered using Procipitate (Ligochem) and selected regions were amplified using Taq DNA Polymerase (Promega). After preheating reactions for 3 min. at 94°C, 30 cycles were run at 94°C for 30 sec., 54°C for 30 sec., and 72°C for 1 min. Regions within pAMH3D were first PCR-amplified using primer pairs PDIA01F-02R, PDIA03F-04R, PDIA05F-06R, PDIA07F-08R, PDIA09F-10R, PDIA11F-12R, and PDIA13F-14R (see Table 1). The amplicons were purified on Qiagen spin columns and sequenced directly without cloning as described [11]. Sequences for regions beyond the ends of AF095730 were generated directly from BAC DNA using outward facing primers (PDIA02R and 03F) derived from the termini of AF095730, followed by additional outward extensions with primers PDIA16F, 17R, 18R, 19R, 20R, 21F (Table 1). BAC clone sequences have been deposited [GenBank: AY656654-AY656662].

### *In silico *methods

Selected regions of AF095730 and their conceptual translations were used to query all relevant Genbank databases, including nr, Genome Sequence Survey (GSS) and Expressed Sequence Tag (EST), with BLASTn and BLASTp searches [44,45]. Conserved protein domains were identified with CDD [46].

For assembly of the consensus nucleotide sequence, *Glycine max *accessions from the GSS and EST databases with bit scores greater than 200 (E values < 10^-52^) were added to the consensus construct. These criteria generally reflect >90% DNA sequence identity over at least 200 bp of overlap. The nr nucleotide database contained no significant hits. New additions to the ends of the expanding consensus were used to re-query the databases until the LTR redundancy was recognized and the contig formed a circle. Contigs were assembled using the Seqman program from Lasergene 5 (DNAStar). To locate the LTR, direct repeats greater than 25 bp were first identified using Lasergene GeneQuest (DNAStar) and the termini of the LTRs were confirmed by manual inspection, as were other non-coding features of the sequence. Because LTR junction sequences at both termini contained either internal element DNA or external flanking DNAs, these were carefully examined for consensus DNA (internal) or unique DNAs (external). External DNAs were trimmed from the contig. Potential splice junctions were evaluated using GeneSplicer [47] and NetGene2 [48]. Transmembrane domains were predicted using TMPred [49]

The *pol *region of the conceptually translated consensus sequence was used to query the Genbank protein database for related sequences. A ClustalW alignment (see Additional file 1) was generated from a contiguous region of RT representing conserved domains two through seven [29] using Lasergene 5 (DNAStar), and a neighbor joining tree using p distances with 1000 bootstrap pseudo-replicates was constructed using MEGA2 [50].

## Abbreviations

LTR: long terminal repeat; RT: reverse transcriptase; prot: protease; env: envelope; PBS: tRNA primer binding site; PPT: polypurine tract; GSS: genome survey sequence; HTGS: high throughput genomic sequence

## Authors' contributions

SY identified, recovered, amplified, and sequenced DNA from BAC library clones and sequenced DNA from the λ library clones. PB identified and recovered DNA from the λ library clones. HL isolated and sequenced DNA from λ library sub-clones and performed all the *in silico *and phylogenetic analyses. HL prepared the manuscript for review by the authors and all authors approved the final draft.

## Supplementary Material

Additional File 1 ClustalW alignment of conserved reverse transcriptase domains for selected plant Ty3-gypsy family retroelements. Amino acid sequence alignments generated by ClustalW using the Megalign program from Lasergene 5 were imported into MEGA2 for phylogenetic analysis. Click here for file
